# Indolepropionic Acid, a Gut Bacteria-Produced Tryptophan Metabolite and the Risk of Type 2 Diabetes and Non-Alcoholic Fatty Liver Disease

**DOI:** 10.3390/nu14214695

**Published:** 2022-11-06

**Authors:** Ratika Sehgal, Vanessa D. de Mello, Ville Männistö, Jaana Lindström, Jaakko Tuomilehto, Jussi Pihlajamäki, Matti Uusitupa

**Affiliations:** 1 Institute of Public Health and Clinical Nutrition, Department of Clinical Nutrition, University of Eastern Finland, 70211 Kuopio, Finland; 2 Department of Medicine, University of Eastern Finland, Kuopio University Hospital, 70211 Kuopio, Finland; 3 Public Health Prevention Unit, Finnish Institute for Health and Welfare, 00271 Helsinki, Finland; 4 Department of Public Health, University of Helsinki, 00014 Helsinki, Finland; 5 Diabetes Research Group, King Abdulaziz University, Jeddah 21589, Saudi Arabia; 6 Department of Medicine, Endocrinology and Clinical Nutrition, Kuopio University Hospital, 70210 Kuopio, Finland

**Keywords:** indolepropionic acid, type 2 diabetes, insulin, NAFLD, diet, gut microbiota

## Abstract

An intricate relationship between gut microbiota, diet, and the human body has recently been extensively investigated. Gut microbiota and gut-derived metabolites, especially, tryptophan derivatives, modulate metabolic and immune functions in health and disease. One of the tryptophan derivatives, indolepropionic acid (IPA), is increasingly being studied as a marker for the onset and development of metabolic disorders, including type 2 diabetes (T2D) and non-alcoholic fatty liver disease (NAFLD). The IPA levels heavily depend on the diet, particularly dietary fiber, and show huge variations among individuals. We suggest that these variations could partially be explained using genetic variants known to be associated with specific diseases such as T2D. In this narrative review, we elaborate on the beneficial effects of IPA in the mitigation of T2D and NAFLD, and further study the putative interactions between IPA and well-known genetic variants (*TCF7L2*, *FTO*, and *PPARG*), known to be associated with the risk of T2D. We have investigated the long-term preventive value of IPA in the development of T2D in the Finnish prediabetic population and the correlation of IPA with phytosterols in obese individuals from an ongoing Kuopio obesity surgery study. The diversity in IPA-linked mechanisms affecting glucose metabolism and liver fibrosis makes it a unique small metabolite and a promising candidate for the reversal or management of metabolic disorders, mainly T2D and NAFLD.

## 1. Introduction

Indolepropionic acid (IPA) is a tryptophan metabolite that is exclusively produced by gut microbiota. It was discovered in 1923 [1], but interest to examine the potential health effects of IPA on type 2 diabetes (T2D) and related diseases has increased not until recent years. IPA has been shown to have neuroprotective properties, including beneficial effects on Alzheimer’s disease [2,3]. IPA can protect the intestinal barrier through pregnane X receptor (*PXR*) activation, and it has been shown to exhibit both anti-oxidative and anti-inflammatory activities [3,4]. Recently, along with the well-known effects of short-chain fatty acids and branched-chain amino acids [5,6], IPA has been linked to glucose metabolism and the risk of T2D [4,7]. 

In our previous studies from the Finnish Diabetes Prevention Study (DPS), we have shown that serum IPA concentrations were associated with higher dietary fiber intake and, that IPA protected against the development of T2D by preserving insulin secretion capacity along the years in individuals with impaired glucose tolerance (IGT) [7,8]. Indeed, it has been found that IPA may have a direct effect on insulin secretion through various mechanisms [9]. This finding is of particular interest since many gut-derived metabolites have been linked with T2D risk, e.g., certain bile acids, short-chain fatty acids, branched-chain amino acids, and numerous lipid metabolites associated with insulin resistance [5,6]. 

Non-alcoholic fatty liver disease (NAFLD) and T2D are often considered as two sides of the same coin in terms of underlying metabolic complexity [10]. Besides modifying and preserving long-term insulin secretion, IPA may also have anti-inflammatory and anti-oxidative properties that might be related to the protective effect of IPA on pancreatic beta-cell as well as hepatocytes [9]. We have shown that serum concentrations of IPA correlated with dietary intake of fiber in pre-diabetic individuals with IGT [7,8]. A higher dietary fiber intake has constantly been shown to protect from T2D and other chronic non-communicable diseases, including cardiovascular diseases and cancer [11,12,13]. Even in the DPS, the prevention effect on T2D was best in the study participants with a high dietary fiber but low-fat intake [14], which is in accordance with the hypothesis that IPA may play a causal role in glucose dysmetabolism, as suggested also by other researchers [15]. 

Although IPA is produced exclusively by gut microbiota [16], its effects on glucose metabolism may be modified by genetic variants that are known to be associated with T2D. Furthermore, gut microbial species that are not directly producing IPA may indirectly be involved in its production [4]. IPA has also been shown to have antimycobacterial activity and in tuberculosis, it may dampen the inflammation process [17] and have beneficial effects on peripheral nerve regeneration [18]. We have also shown previously that serum IPA levels in obese individuals with liver fibrosis undergoing bariatric surgery from Kuopio Obesity Surgery (KOBS) study were reduced compared to obese individuals without liver fibrosis. Moreover, we identified a negative association between fasting plasma glucose levels and IPA levels in the KOBS cohort [19]. 

In the present narrative review on IPA and T2D and fatty liver disease, we will extend our analyses on the available IPA data that we have published earlier to explore the putative interactions between IPA and well-known genetic variants (*TCF7L2*, *FTO*, and *PPARG*) that were associated with the risk of T2D in DPS study participants [20]. Next, we will discuss the potential protective value of IPA in the prevention of metabolic disorders, inclusive of T2D.

## 2. Diet, Gut Microbiota, and Indolepropionic Acid

### 2.1. The Role of Gut Microbiota in the Formation of IPA

Tryptophan is an essential amino acid coming from dietary sources [21]. It is metabolized by the liver through the serotonergic pathway to serotonin and melatonin or through the kynurenine pathway to four metabolites, kynurenine, kynurenic acid, anthranilic acid, and quinolinic acid [3]. The qualitative or quantitative alterations in the gut microbiota can intricately modulate human physiology as well as pathophysiology resulting in obesity, T2D, non-alcoholic fatty liver disease (NAFLD), metabolic syndrome, inflammatory bowel disease, cardiovascular diseases, and neurological disorders [22]. Besides the endogenous metabolism, it has been shown that certain gut bacteria are capable to synthesize IPA from tryptophan through the indolic pathway [23]. *Clostridium* (*Cl.*) *sporogenes* and *Cl. botulinum* but also *Peptostreptococcus anaerobius* and three strains of *Cl. calaveris* may also synthesize IPA [16]. In fact, *Cl. sporogenes* supplementation in mice has been reported to significantly elevate IPA levels and reduce the kynurenine production in the muscle tissue, while the circulating IPA levels were not found to be increased [24]. Also, *Lechevalieria aerocolonigenes* via tryptophan deamination using amino acid oxidase might lead to IPA synthesis [25]. It has been reported that IPA production in the gut is mainly regulated by tryptophan aminotransferase [23,26] and might also be dependent on bacterial tryptophanase [27]. Other than these, bacterial protein caseinolytic peptidase B protein homolog also associates positively with plasma IPA levels in humans, and more importantly, the same protein homolog is negatively associated with anthropometric measures of obesity, such as total fat mass, body mass index (BMI), and waist circumference [28]. The importance of IPA in humans can be understood from a study with two cohorts of genotypically human leukocyte antigen-identical related organ recipients and donors [29]. In these individuals, the onset of acute graft versus host disease after the transplant was found to have the highest significant variation in IPA levels, which in turn has been proposed to induce the indoleamine 2-3-dioxygenase pathway in immune cells [29]. 

Microbially produced IPA in the intestinal tract can easily be absorbed by the epithelial cells where it can serve as a ligand for both *PXR* and aryl hydrocarbon (*AhR*) receptors [30]. IPA can then diffuse into the peripheral and portal blood circulation and gain direct access to multiple body organs [31]. The gut-blood barrier permeability to specific substances including IPA has been tested in the past using fluorescein isothiocyanate-dextran assay in mice [32,33]. It plays a significant role in the gut by fortifying intestinal permeability and has also been shown to affect systemic immunity. It has been shown that this effect might be directly related to the *PXR* activation by IPA [32]. Treatment of myoblasts with IPA effectively protected them against lipopolysaccharide-induced inflammation via *PXR* activation and in turn, restored the inhibited miR26a23p (novel muscle inflammation regulatory factor) expression [24]. IPA is often considered a critical marker of properly functioning gut microbiota [34]. IPA is a biomarker of active ulcerative colitis and for disease, remission based on a study in human patients with intestinal bowel disorder [35]. Interestingly, IPA may play a role in the protection of the gut from chronic inflammatory bowel diseases, traumatic colon injury, and also colitis [3,36,37]. As a result of a strong link between the gut and other metabolically active organs including the liver, IPA plays an important role in the pathogenesis of NAFLD and T2D [38]. This link has also been found to be extending to the brain as it has been proposed that IPA treatment could even reduce T2D-linked cognitive decline in addition to Alzheimer’s disease and multiple sclerosis [39,40]. 

**Figure 1:**
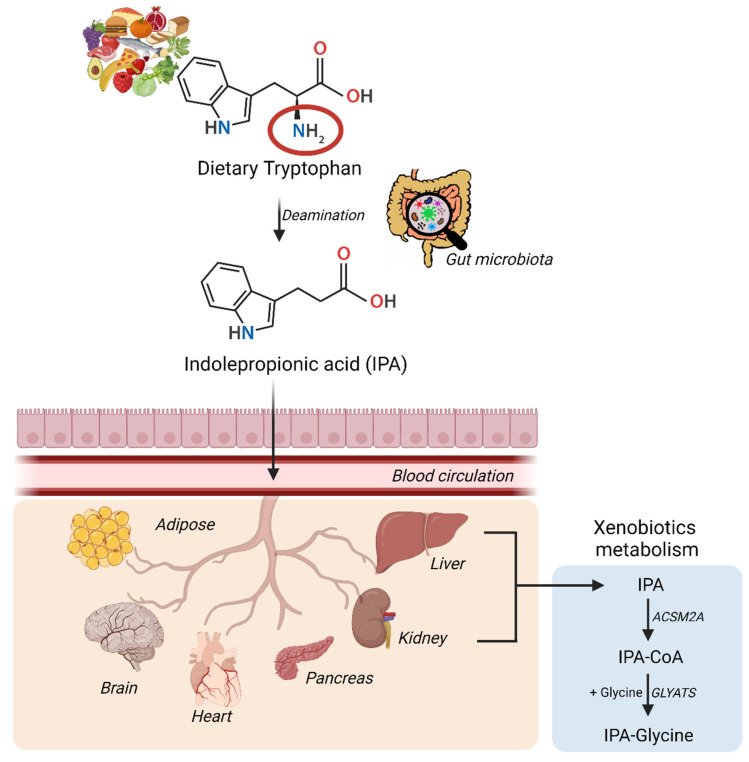
Synthesis and conversion of indolepropionic acid in humans. Dietary tryptophan is deaminated via the gut microbiota into IPA, that then enters the blood circulation directly from the gut epithelium. IPA via the circulation gains access to multiple organs, ultimately ending up in liver or kidney to be metabolized and excreted out of the body. *ACSM2*—Acyl-CoA synthetase medium chain family member 2A; *GLYATS*—Glycine-N-acyltransferase.

The synthesis and conversion of IPA in humans have been summarized in the Figure 1. IPA via the blood circulation can gain excess to multiple organs either via passive diffusion or with the help of amino acid transporters and can induce multiple beneficial effects [41]. Specifically in the liver and kidney, IPA can undergo xenobiotic metabolism to be excreted out of the body as a glycine conjugate [42]. IPA concentrations as high as 100 µM have shown no hepatotoxic effects as measured using cell viability assay [19]. However, in other cell types such as cardiomyocytes, IPA concentrations as high as 1 mM are also well tolerated [43]. In the clinical trial (NCT01898884) targeted to study the pharmacokinetic-pharmacodynamic profile of IPA as a treatment option for Friedreich’s ataxia reports that single dose of 450 mg orally under fasting conditions of IPA leads to treatment-emergent adverse events but no serious adverse events for doses up till 1200 mg.

### 2.2. Diet and Indolepropionic Acid

**Figure 2:**
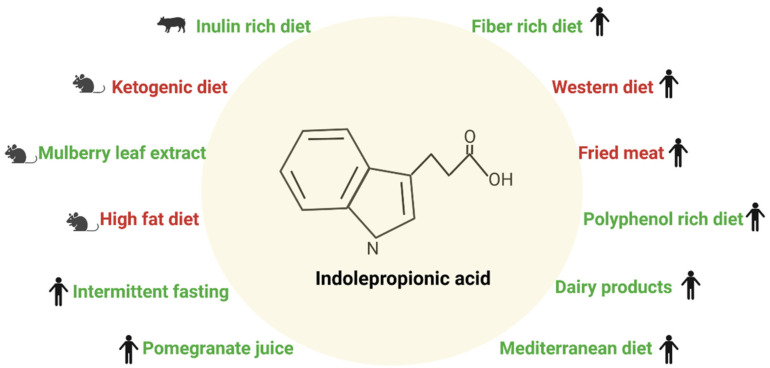
Effect of a specific type of diet on circulating indolepropionic acid levels. The green color text means the IPA levels were found to be increased and had a beneficial effect and the red colored text means the levels were reduced and were harmful. The symbols in the figure represent the model system (mouse, pig, or human) used for studying the effects of IPA.

Circulating levels of IPA are highly variable due to diet and the composition of gut microbiota [4]. Among the various food items, dietary fiber has been shown to protect people from many chronic diseases, including cardiovascular diseases, some cancers, and T2D [11,12,13]. Modern “westernized” diet pattern is particularly low in dietary fiber and, along with the obesity epidemic, low fiber intake contributes to an increased incidence of T2D worldwide [12]. In our studies using non-targeted metabolomics, we found that serum concentrations of IPA were associated with higher fiber intake and a lower risk of T2D, and better insulin secretion [7,8]. This finding suggests that IPA, at least in part, may mediate the preventive effect of dietary fiber on T2D risk and metabolic disorders by modifying gut microbiota enrichening the bacteria that are producing IPA locally, and increasing its concentrations in the bloodstream. Figure 2 summarizes the effect of a specific type of diet on circulating IPA levels based on studies described in the following paragraphs.

A recent study based on the data from 1018 middle-aged women from the TwinsUK cohort showed that serum IPA levels were correlated with a higher diversity of gut microbiota explaining 20% of the variation of serum IPA, whereas nutritional and host genetic variables explained only 4% of this variation [4]. In this study, the correlation of IPA with fiber intake (estimated intakes of essential fatty acids and fiber calculated as grams/day based on the UK nutrient database) was confirmed. Furthermore, IPA’s protective effect on metabolic disorders was evaluated, and for the first time, IPA’s connection with visceral fat mass was reported in a large study population. Recently, IPA was associated with a lower risk of T2D in a study with five different cohorts including altogether 9180 study participants. In this study, intake of tryptophan or a protein-rich diet had no relationship with IPA, but a diet rich in dietary fiber showed a direct association with IPA. Furthermore, this relationship was partially explained by the gut bacteria *Firmicutes*, known to metabolize fiber. Serum levels of IPA were also found to associate with the activity of lactase enzyme and were observed only in individuals with non-persistent lactase activity [44]. There has been a lot of interest in probiotics that may modify gut microbiota and enrich those bacteria producing e.g., IPA, but no definite progress regarding the protective effect of IPA and the role of probiotics has been reached so far [45,46].

Besides dietary fiber, there may be other dietary factors that may increase or decrease IPA production by gut microbiota. Mediterranean-type diet in humans [47] and mulberry leaf extract consumption in mice [48] have been shown to increase IPA concentrations, whereas fast food diet decreased its production in the gut [47]. Most of these changes in IPA can be attributed to fiber intake from the diet. In mice, oat and rye fiber-rich diets compared with the western diet resulted in an increase of short-chain fatty acids and changed tryptophan metabolism towards lower production of serotonin. Instead, tryptophan diversion from the serotonin synthesis pathway to microbial indole production was suggested to increase that ultimately leads to stimulated IPA production. Unfortunately, IPA was not directly measured in this study, but the authors speculate that fiber-rich diets alter gut microbiota in a way that may affect tryptophan metabolism and at the same time have anti-inflammatory properties and beneficial effects on glucose metabolism [49]. Additionally, plasma IPA levels were reported to be significantly reduced in ketogenic diet-fed animals versus normal diet-fed littermates [50]. 

In humans, intake of fried meat resulted in higher concentrations of many inflammatory factors (lipopolysaccharide, TNF-α, interleukin (IL)-1β, and IL-10) and interestingly lower fecal content of IPA [51]. Fried meat intake directly affected the microbial composition with marked changes in microbial community richness and lower abundance of *Lachnospiraceae* and *Flavonifractor*, which was significantly correlated with the decreasing fecal IPA content [51]. Inulin-rich diet in pigs has been shown to increase serum IPA concentration and it resulted in a lowered concentration of branched-chain amino acids [52]. In a randomized crossover study in healthy men, the post-prandial serum IPA levels were found to be different between milk and yogurt intake groups, with IPA levels lower in men in the yogurt group compared to the milk group [53]. Also, IPA levels were found to be decreased in the late phase in individuals on an anti-inflammatory dietary mix (consisting of fish oil, green tea extract, resveratrol, vitamin E, vitamin C, and tomato extract) subjected to a postprandial challenge test. These changes were with respect to individuals only subjected to postprandial challenge test (standardized 500 mL dairy shake containing respectively 59, 30, and 12 energy % lipids, carbohydrates, and proteins, respectively) [54]. In a 1-year randomized, double-blind, placebo-controlled trial of pomegranate juice, plasma IPA levels directly correlated with the relative proportion of *Roseburia*, *Lachnospira*, *Sutterella*, *Proteobacterium*, *Collinsella*, and *Actinobacteria* and negatively with *Campylobacteraceae* in healthy adults [55].

A polyphenol-rich diet along with increased fiber intake elevated serum concentrations of IPA in older individuals with normal renal function, but not in those with impaired renal function. In this study, serum IPA was also inversely correlated with serum C-reactive protein and IL-6 levels [56], in line with our findings [7,8]. Obesity was associated with marked lower serum IPA levels [4,57], and with lowered local colonic concentrations of IPA in mice [57]. Furthermore, the administration of IPA had beneficial effects on weight, glucose, and lipid metabolism in mice [57]. IPA has also anti-inflammatory effects on adipose tissue, and locally in the gut, it improved gut integrity [57]. In obese people, the serum concentration of IPA was found to be decreased, but an increase was reported after bariatric surgery [33]. Furthermore, IPA may modify addiction to food at the brain level [58]. Surprisingly few studies have dealt with the effects of type and quality of diet, including dietary fiber, on IPA production so far.

## 3. Indolepropionic Acid and Type 2 Diabetes

### 3.1. Effect of Indolepropionic Acid on Glucose Metabolism and Insulin Secretion

The gut microbiome and derived metabolites are known as modifiable environmental factors, playing a major role in the development of T2D. It is highly likely that gut-derived IPA directly regulates key mechanisms linked with T2D pathogenesis [5]. For instance, in the DPS, people who developed T2D had significantly lower serum concentrations of IPA many years before the onset of T2D than those who did not develop T2D during a 15-year follow-up period [7]. In those who did not develop T2D, IPA was associated with the preservation of beta-cell function [7]. 

To address the same question, Gou et al. developed a novel interpretable machine-learning algorithm with data from large-scale human cohorts [59]. The results from this approach were further confirmed using fecal microbiota transfer experiments. IPA was among the six metabolites that were found to be negatively correlated with the microbiome risk score for T2D (derived from three independent cohorts based in 9111 Chinese participants) [59]. In a large-scale study (*n* = 789 with and *n* = 2127 without T2D from the innovative medicines initiative- diabetes research on patient stratification cohort), serum IPA levels were found to be correlated with blood transcriptomic module, identified using a weighted gene co-expression network analysis approach [60]. This transcriptomic module is specifically associated with insulin resistance and glucose intolerance in this study [60]. The most recent systematic review and meta-analysis of prospective cohort studies (with 71,196 participants among which 16% had T2D) identified that higher levels of IPA were associated with a lower risk of T2D [61]. The summary relative risk for the association between one standard deviation increase in levels of IPA and risk of incident T2D was 0.82 (95% confidence interval 0.74–0.92) [61]. The percent of the variation in IPA levels due to heterogeneity rather than chance across the studies included in the meta-analysis was 67% [61]. These results reinforce the findings from other studies that IPA levels in people are highly variable and hence might not be the best biomarker candidate. In addition, circulating IPA was found to be positively associated with IL-6 levels, a marker of inflammation in functionally limited older adults [62]. These findings are contrasting with our findings, however, the population in this study is very different from the DPS cohort. 

Qi et al. investigated five epidemiological cohorts to study the association between tryptophan metabolites in blood circulation and incident T2D (*n* = 9180). They found that IPA was inversely associated with the incidence of T2D in models with adjustments for obesity measures [44]. In a small cohort of Caucasian women (*n* = 25), targeted metabolome analysis revealed negative IPA with body weight and BMI in obese (with and without T2D) individuals [63]. In a cross-sectional study of the metabolic syndrome in men (METSIM) study with a 7.4-year follow-up, including 5181 Finnish men, fasting plasma IPA was found to be nominally associated with decreased risk for T2D in models adjusted for confounding variables [64]. Also, during a 7.4-year follow-up, IPA was inversely associated with fasting plasma glucose (*p*-value < 0.01) and 2-h plasma glucose (*p*-value < 0.05), while a direct association was observed with disposition index (*p*-value < 0.05) and insulin secretion (*p*-value < 0.01) [64]. Another study using the METSIM participants (*n* = 8749), concluded a direct association between IPA levels and physical activity [65]. Physical activity was assessed with a self-reported questionnaire; increased physical activity was independently found to be associated with a lower risk of T2D [65]. Additionally, IPA along with other indole derivatives has also been found to be inversely associated with food addiction, a major contributing factor in the onset and progression of lipid and glucose dysregulations [58]. Plasma IPA levels in a cross-sectional case-control study have been reported to be elevated in diabetic patients with or without nephropathy compared with controls [66]. The researchers concluded that these elevated levels are likely due to altered microbiota response to medications for lowering glucose levels [66]. 

The mechanistic effect of IPA on glucose metabolism has not yet been studied in detail. One of the first studies in 2018, assessed in rats the effect of 6-week IPA dietary supplementation (27.3 mg/Kg/day) compared to the chow diet. Fasting plasma glucose and insulin were reduced in rats fed with IPA [15]. The results also showed no changes in the elevated plus maze (a measure of anxiety), open field (measure of locomotor activity, and exploration habits), and forced swim test (evaluates depressive state and behavioral despair). Additionally, no changes were recorded in plasma metabolic hormones in rats supplemented with IPA [15]. These results propose that IPA might directly regulate the uptake or production of glucose, thereby affecting glucose metabolism. Moreover, the direct effect of IPA on glucose/fructose transporter (*GLUT-5*) mRNA levels has been identified using an in vitro model [33]. Gundu et al. studied the effect of a 2-week IPA supplementation in streptozotocin-induced diabetic rats [67]. They reported that IPA treatment reduced oxidative damage, endoplasmic reticulum stress, mitochondrial function, and apoptotic markers in the diseased group with changes in pain behavior as well [67]. Kelly et al. have developed a panel of metabolites (Xenoscan) to detect xenometabolites and they found IPA to be negatively correlated with species within the *Oscillibacter* genus in the University of California, Davis-T2D rats that are considered to be models for individuals who are prediabetic or have recently been diagnosed with T2D [68]. Moreover, intake of a tryptophan-enriched diet may have a beneficial effect on insulin sensitivity via IPA’s activity as a *PPAR*α/γ co-agonist and a potential drug for the reversal of insulin resistance [69].

The studies that have identified mechanisms which might be directly or indirectly related to glucose metabolism using in vivo or in vitro models have been summarized in Table 1. Briefly, IPA has been found to directly affect mitochondrial function, expression of anti- and pro-inflammatory cytokines, and genes regulating lipid biosynthesis, glucose uptake, and metabolism [17]. In fact, how this association of IPA with T2D and glucose metabolism is affected by the presence or absence of T2D-specific genetic variants has also not been fully explored.

### 3.2. Interaction of IPA with T2D-Linked Genetic Variants

Over the last decade metabolite, genome-wide association studies have unfolded novel associations between genetic variants and metabolic phenotypes in an elaborative manner. This is a step towards precision nutrition. Koshiba et al. performed plasma non-targeted metabolomics in a Japanese prospective cohort (*n* = 1008). They identified a genetic variation rs59261767 in acyl-coA-synthase 2A (*ACSM2A*), a mitochondrial enzyme involved in fatty acids and xenobiotics metabolism to be linked with IPA at a genome-wide significant *p*-value threshold [42]. The homozygotes (*n* = 31) were found to have almost five-fold increased median IPA concentration compared to wild-type homozygotes (*n* = 672), while the heterozygotes (*n* = 305) had only a 1.7-fold elevation in IPA concentrations. Independently, IPA was also reported to be associated with another single nucleotide polymorphism (SNP; rs1394678) for *ACSM2A* [71]. In an extended study, overall variation in circulating IPA levels was found to be contributed by variant rs1394678 in *ACSM2A*, dietary factors, and gut microbiome factors [4]. IPA was also found to be associated with obesity and other cardiometabolic traits, which are also independent risk factors for T2D development. The researchers have identified 10.7% (standard error = 5.8%) genome-wide SNP-based heritability for IPA in the selected cohorts and a genetic causality proportion of 76% (*p*-value < 0.01) with T2D. As expected, increased IPA levels in a sub-population from the same study (*n* = 3938) were also found to be associated with higher intakes of whole grains, legumes, vegetables, fruits, and nuts and lower intakes of refined grains and red meat. IPA production in the gut lumen has also been proposed as a potential mechanism that is reflected as *Bifidobacterium* and T2D association in human studies [56]. Qi et al. also identified variant (rs4988235) in the *LCT* gene as a novel locus for IPA as well as *Bifidobacterium* in a genome-wide association study [44].

**Figure 3:**
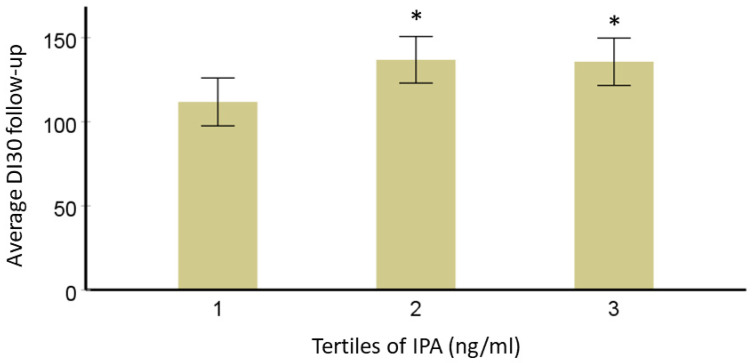
Averaged disposition index during the 7-year follow-up (*y*-axis) by tertiles (1st *n* = 31, 2nd *n* = 32, 3rd *n* = 31) for serum IPA concentration (*x*-axis, models adjusted for age, sex, BMI, and study group) in the DPS. * Represents *p*-value < 0.05 vs. 3rd one for IPA. The bars represent the estimated marginal means with confidence intervals (95%).

We have previously identified a direct relationship between serum IPA level and T2D risk. In this narrative review, we further explored if T2D-specific genetic variants (*TCF7L2* rs7903146 and rs12255372; *FTO* rs9939609 and *PPARG* rs1801282) from the same DPS population modified this association [72,73,74]. A total of 522 individuals with IGT were originally randomly allocated into either a lifestyle intervention or control group for a mean four-year intervention (active study) period. Data from the post-intervention follow-up that was carried out with annual examinations (body composition, glucose levels, insulin levels, and disposition index_30_-DI_30_) was available for this study [75]. DI_30_ is the estimate for beta cell function or peripheral insulin sensitivity, as validated before [76]. Mathematically, DI_30_ = Matsuda insulin sensitivity index × ratio of total insulin area under the curve (AUC) and total glucose AUC during 0–30 min of the oral glucose tolerance test. After data normalization, analysis of variance models adjusted for study group, age, sex, and BMI were used to study the associations of IPA with long-term follow-up insulin secretion (DI_30_), fasting, and postprandial glucose or insulin additionally considering the effect of *TCF7L2*, *FTO* or *PPARG* genotypes (categorical variable; dominant model). For testing correlations, we applied Pearson’s correlation test and the value of *p*-value < 0.05 was considered significant. In models adjusted for age, sex, BMI, and study group, persons in the lowest third for serum IPA concentration have lower averaged DI during the 7-year FU compared with those in the second and third categories (Figure 3). This association did not change by *TCF7L2* variants rs7903146 and rs12255372, which are known to be associated with insulin secretion [77], which was also the case in the DPS population [72]. Another variant associated with the development of T2D in the DPS, *PPARG* rs1801282 may be associated with improvements in insulin sensitivity [74]. When this variant was included in the model, the association of IPA with DI_30_ was not lost. Also, the interaction of variant rs1801282 with IPA was not significant. 

**Figure 4:**
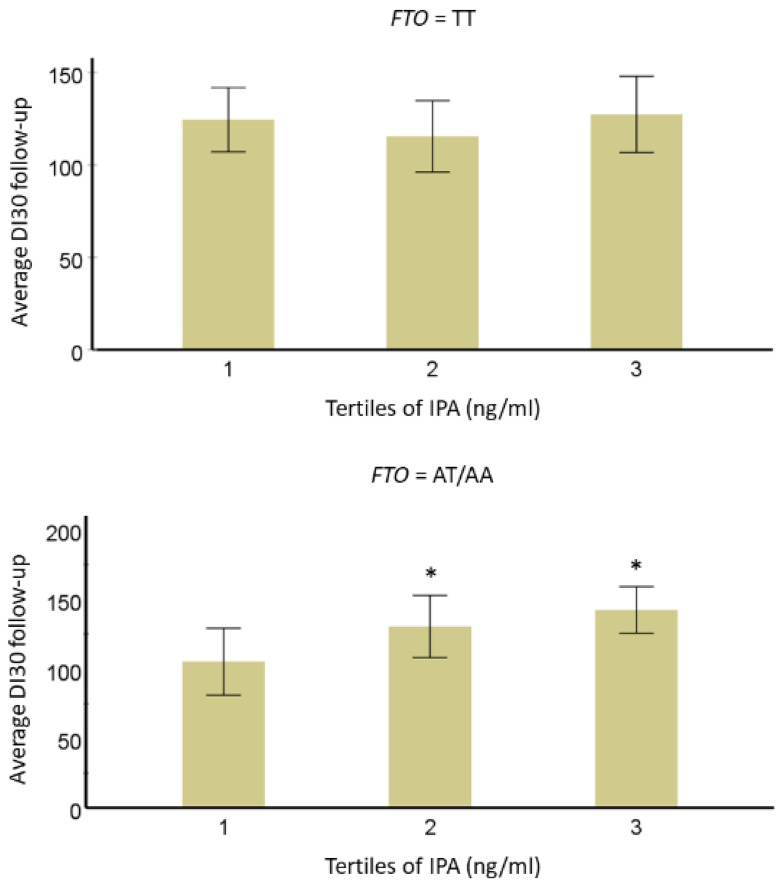
Averaged disposition index during the 7-year follow-up by third for serum IPA concentration (models adjusted for age, sex, BMI, and study group) divided based on the presence of major or minor allele of *FTO* in the DPS. * Represents *p*-value < 0.05 vs. 3rd one for IPA. The bars represent the estimated marginal means with confidence intervals (95%).

We observed a significant trend for the interaction of IPA with *FTO* variant rs9939609 (*p* = 0.05). In individuals with the minor allele A, we found that those in the lowest IPA tertile had lower averaged IPA concentrations as compared with the second (*p*-value = 0.003) and third (*p*-value = 0.018) tertiles for DI_30_. No association was found in individuals with a major T allele (*p*-value = 0.98) (Figure 4). This interaction of IPA with *FTO* suggests a novel mechanism that might include an impact linked to food cognition and addiction as suggested by Dong et al. [58].

**Figure 5:**
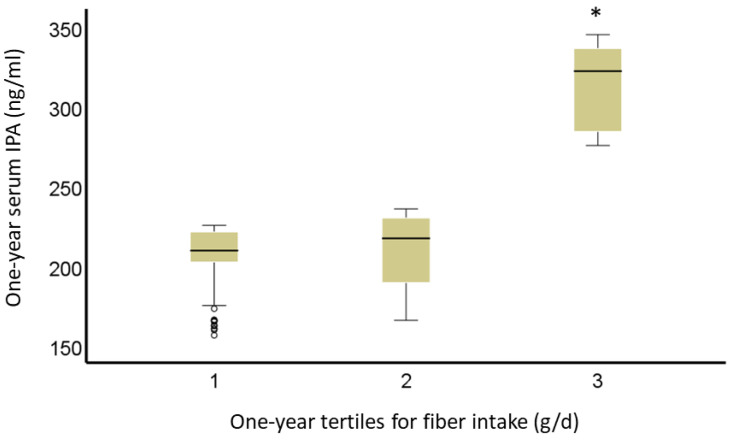
Serum IPA (ng/mL) by tertiles of fiber intake (g/d) at year 1 in the DPS. * Represents *p*-value < 0.05 vs. 3rd. The bars represent the estimated marginal means, and the whiskers are confidence intervals (95%).

We also investigated whether thirds of serum IPA concentrations would be related to other surrogates of serum insulin and plasma glucose metabolism (fasting and post-challenge 1 h and 2 h glucose and insulin concentrations). As with DI, follow-up 1-h and 2-h post-challenge glucose levels during an oral glucose tolerance test were associated with thirds of IPA in a 1-year study. Participants in the lowest third of serum IPA concentration have lower 1-h and 2-h glucose levels during the 7-year follow-up compared with the second and third (*p*-value = 0.013, and *p*-value = 0.03, respectively), but no significant differences between those in the highest third vs. the rest (*p*-value < 0.10 for all comparisons). We were able to confirm, in all participants, an association between fiber intake and IPA. People in the lowest third of fiber intake (13.5 ± 2.5 g/d) have a significantly lower serum concentration of IPA compared with those in either 2nd (*p*-value < 0.001) or 3rd (*p*-value = 4 × 10^−6^) third of fiber intake (19.7 ± 1.5 g/d; 28.7 ± 5.3 g/d, respectively). There was no significant difference in IPA serum concentration between the 2nd and 3rd third of fiber intake (*p*-value = 0.49) (Figure 5).

## 4. Indolepropionic Acid and Non-Alcoholic Fatty Liver Disease

The gut-liver axis acts as an important bridge in the onset and progression of diverse liver disorders including NAFLD, non-alcoholic steatohepatitis (NASH), and hepatocellular carcinoma [78]. The metabolic dysregulations leading to these hepatic alterations are closely linked to the pathogenesis of T2D and cardiovascular diseases [79]. Existing evidence links gut-derived indole and indole derivatives to liver health and integrity [80]. As part of the ongoing Kuopio obesity surgery (KOBS, since 2005, ongoing > 500 obese participants with liver biopsies) study that investigates especially nutrient-related metabolites and their association with histologically characterized NAFLD and related metabolic diseases, such type 2 diabetes, we identified circulating levels of IPA to be associated with liver lobular inflammation and fibrosis in obese individuals (*n* = 233). We further reported, an association of IPA with 278 liver transcripts, that were found to be enriched in pathways involved in the regulation of hepatic stellate cell activation, the main mechanism triggering liver fibrosis. Moreover, we highlighted the capability of IPA to partially rescue this activated phenotype using transforming growth factor (TGF)-β1-activated LX-2 cells by directly affecting cell adhesion, cell migration, and mRNA expression of genes involved in extracellular matrix formation and function [19]. In fact, we have also shown that serum IPA levels measured using non-targeted metabolomics in obese individuals awaiting bariatric surgery from KOBS were lower in ones who have fibrosis compared to ones without liver fibrosis. These findings had a significantly lower *p*-value if only individuals without T2D were considered for the analysis [19]. Additionally, the serum IPA level was associated with fasting plasma glucose. These results indicate that IPA and glucose metabolism seems to be closely related, especially in the presence of obesity and NAFLD.

Tryptophan, a substrate for IPA, is derived from dietary sources as are the phytosterols. Both are known for their cholesterol-lowering effects [81]. For this narrative review, we explored the correlation between these phytosterols and IPA in KOBS individuals (*n* = 48). We found a direct correlation between IPA and phytosterols; sitosterol (r = 0.348, *p*-value = 0.016) and campesterol (r = 0.359, *p*-value = 0.012). However, no correlation was found between IPA and average serum total cholesterol or IPA and the above-mentioned phytosterols in the DPS. Overall, these findings suggest that IPA may also have a role in the dietary regulation of cholesterol metabolism. Because of the known link between cholesterol metabolism and NAFLD [82], this may also contribute to the link between IPA and NAFLD.

Previously, it has been shown circulating IPA level increase after bariatric surgery in individuals with T2D [33]. These results indicate an active involvement of gut microbiota not only in the pathogenesis of metabolic diseases but also in their recovery. Circulating serum metabolites in normal-weight, overweight, and obese Mexican American children were measured to identify novel biomarkers for childhood obesity-related traits. In this study, IPA levels were found to be significantly lower in overweight and obese children compared with normal-weight ones. Moreover, the IPA level was inversely correlated with BMI and waist circumference and directly with high-density lipoprotein-cholesterol [83]. This suggests that IPA is also related to obesity-related metabolic complications also in children. IPA and other tryptophan derivatives have also been found to be associated with cardiovascular events, a common co-morbidity in NAFLD, NASH, and T2D [84,85]. In a recent study, the *fldAIBC* gene cluster, responsible for IPA metabolism in humans, was found to be less abundant in patients with coronary artery disease (*n* = 60). In the same study, IPA was significantly associated with the risk of atherosclerosis, independently of other risk factors [84]. Accordingly, in a targeted metabolomics study (*n* = 22 control and *n* = 100 advanced atherosclerosis), plasma IPA concentration was significantly lower in atherosclerotic individuals when adjusting for traditional risk factors [85].

IPA is well known for its hydroxyl radical and lipid peroxide scavenging activity. Lefler et al. investigated the capacity of IPA to ameliorate liver damage following an ischemic event in rats [86]. They found that the pretreatment with IPA significantly reduced serum alanine aminotransferase, and aspartate aminotransferase during the reperfusion [86]. The hepatoprotective effects of IPA have also been reported in rodent studies focused on NAFLD and NASH and in vitro studies related to hepatocyte functioning [87,88]. Research by Zhao et al. identified that the administration of IPA attenuated steatohepatitis in rats with a marked reduction in fibrotic and pro-inflammatory gene expression [87]. IPA treatment successfully restored pathways related to nutrient and energy metabolism in these animals suggesting the potential benefit of IPA on metabolic activity mediated through gut microbiota. The inflammation score and ballooning score along with liver transaminases were also found to be reduced after IPA treatment in the NASH group. Eight weeks of IPA treatment also inhibited lipopolysaccharide-induced protein phosphorylation of *p65*, *IκBα,* and *IKKα/β* along with mRNA levels of *TNFα, IL-1β, IL-6, CCL2, CCR2, TGFβ, αSMA, CTGF, Col1α1, Col1α2*, and *Col3α1* in the livers of high fat diet-fed rats. These results highlight the therapeutic potential of IPA in the treatment of NASH with a specific effect on markers of liver inflammation and fibrosis [19,87]. Even though there are no studies that have examined the relationship between IPA and dietary fiber in the case of NAFLD, a link between fiber consumption, microbiota, and NAFLD already exists [89]. Based on this it would not be inaccurate to propose that gut-derived IPA which is associated with dietary fiber in humans might be relevant for the prevention and treatment of NAFLD and NASH.

The anti-inflammatory effect of IPA has been found in many studies. IPA was found to regulate low-grade inflammation by directly affecting mitochondrial oxidative stress activity and ameliorating cancer cell proliferation [43,90]. In high-fat diet-fed mice, the IPA level was reduced with marked changes in mRNA expression of genes involved in the *AhR-Pemt* signal axis [91]. However, these changes were observed only in non-antibiotic treated mice, signifying the role of gut microbiota in the production of IPA. Consistently, mice fed with the high-cholesterol diet for 14 months had a lower circulating IPA level compared with mice on the control diet [92]. Additionally, it was found that treatment of animals on high-cholesterol diet with atorvastatin potentially improved NASH severity and restored NASH-linked gut microbiota dysbiosis. These changes in turn also significantly elevated the serum IPA levels, reestablishing the strong link between gut microbiota, liver diseases, and IPA. Same researchers also found that IPA treatment to human immortalized hepatocyte cells could lower the cholesterol-induced lipid accumulation in these cells and could potentially reduce cell proliferation in NASH-hepatocellular carcinoma cell lines [92]. These findings suggest that there is a close link between IPA and cholesterol metabolism. However, if increased cholesterol lowers IPA or if IPA changes cholesterol metabolism remains an open question for further investigation. Even in germ-free mice, the IPA level was reduced and this was directly correlated with the increased risk of liver steatohepatitis and tumorigenesis [92]. It has also been proposed that IPA deficiency in germ-free mice might lead to increased susceptibility of hepatocytes to oxidative stress and cell death [93]. 

**Figure 6:**
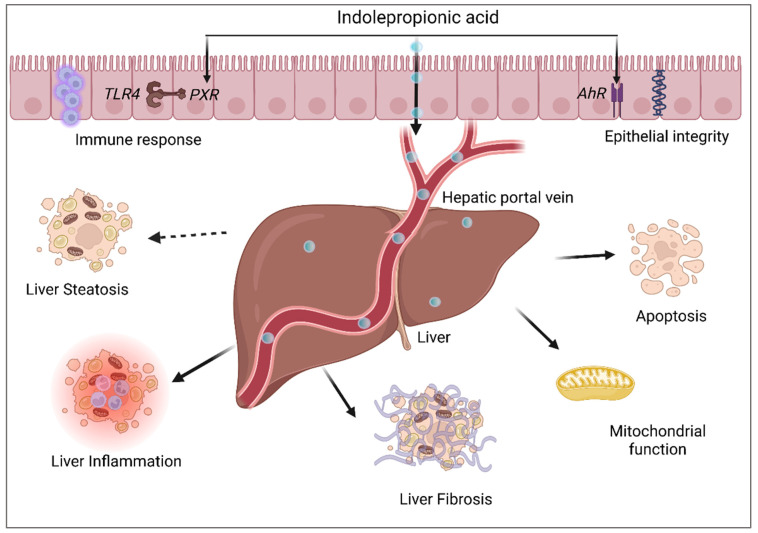
Overview of beneficial effects of IPA on intestinal dynamics and NAFLD. IPA can serve as a ligand for *AhR* and *PXR* in the intestinal epithelial cells leading to enhanced epithelial integrity and immune response (via *TLR4*), respectively. IPA can gain access to the liver directly via the hepatic portal vein and has been shown to be actively involved in liver inflammation, fibrosis, mitochondrial function, and apoptosis.

Pesticide-induced hepatorenal toxicity in rats was found to be rescued after 14 days of treatment with IPA [94]. IPA was able to significantly restore hepatorenal activity, DNA damage, apoptotic response, antioxidant/pro-oxidant, and pro-inflammatory/anti-inflammatory balance along with hepatorenal histological changes [94,95]. Serum hepatic transaminases were also found to be reduced in the IPA-treated rats in a dose-dependent manner, pointing towards IPA’s potential as a hepatoprotective molecule [94]. Ren et al. performed serum and liver metabolomics of dimethylnitrosamine-induced spontaneous hepatocellular carcinoma mice and reported serum IPA levels to be the most strongly associated with the progression of hepatocellular carcinoma [96]. Additionally, IPA might be protective against indoxyl sulfate-induced tubular damage in the kidneys by reducing the expression of fibrotic and inflammatory genes [97]. Many studies have highlighted the beneficial effect of IPA on the liver (Figure 6), however, the mechanism underlying the diverse effects of IPA has not been studied.

## 5. Conclusions

Currently, we can surely conclude that the discovery of the link between gut microbiota and gut-derived metabolites, and in particular IPA, has opened new approaches regarding prevention and treatment options of T2D, metabolic syndrome, and NAFLD. The biological effects of IPA have not only provided new therapeutic options but have increased the knowledge to understand the complex alliance between diet, gut microbiota, and the risk of cardio-metabolic disorders. Many interactions of IPA with various biological processes have already provided new data on the beneficial effects of IPA on intestinal integrity, neuronal function, oxidative stress, lipid metabolism, glucose metabolism inclusive insulin secretion, inflammation, liver injury, and cellular damage. More specifically, a strong relationship between IPA and T2D and NAFLD paves the way for strategies for developing IPA as potential preventive and treatment options for these disorders. In particular, the role of dietary fiber in the prevention of metabolic diseases is important to be addressed seriously. These results demand future research projects focusing on a deep understanding of the mechanisms of action of IPA specifically in cardio-metabolic disorders, yet not forgetting the important role of diet in the prevention of these diseases.

## Figures and Tables

**Table 1:** Beneficial effects of indolepropionic acid in type 2 diabetes and associated diseases.

Disease	Experimental Model	Result	Molecular Mechanism	Reference
T2D-induced cognitive impairment	db/db mice; i.p. IPA (10 mg/Kg/day)-2 weeks	Improved insulin sensitivity and mitochondrial biogenesis	Energy metabolism via mitochondrial function	[39]
Obesity	High-fat diet mice p.o. IPA (100 mg/Kg) from week 7 to 14 after a high-fat diet and NIH3T3 cells (IPA 100 µM)	Reduced body weight, increased energy expenditure, glucose clearance, and insulin sensitivity, and reduced lipid accumulation in serum and liver	Reduced *TNFα*, *TLR4* expression in adipocytes, Increases *IL-25* production in tuft cells via *FFAR3* signaling	[57]
Obesity	Sprague-Dawley i.p. IPA (30 mg/kg) for 1 week	Reduced body weight gain	Not proposed	[70]
Hyperlipidemia	ICR mice p.o. IPA (100 mg/kg) for 60 days	Reduced body weight gain and serum lipids	Reduced expression of *SREBP1c*, *FAS*, *SREBP2*, and *HMGR*	[48]
T2D and obesity	DIO mice IPA (20 mg/kg) for 4 days and T84 cells (IPA—5 µmol/L)	Increased intestinal permeability and reduced cytokine-induced changes	Reduced *GLUT5* in both and *ALDOB* gene expression in mice	[33]
Insulin resistance	Sprague-Dawley rats IPA in diet (27.3 mg/Kg/day) for 6 weeks	Reduced glucose, insulin, and HOMA-IR levels	Glucose uptake	[15]

## Data Availability

Not Applicable.
